# Evaluating Worldwide Disparities in Bladder Cancer Clinical Trial Availability

**DOI:** 10.3390/cancers18111730

**Published:** 2026-05-26

**Authors:** Koral U. Shah, Daniela V. Castro, Xiaochen Li, Miguel Zugman, Salvador Jaime-Casas, Vitor Abreu de Goes, Peter D. Zang, Skylar Reid, Teebro Paul, Jaya Goud, Samuel Dickter, Lea Dickter, Lily Lau, Ruchi Agarwal, Aaron Lee, Nasr Chaudhary, Hedyeh Ebrahimi, Benjamin Mercier, Nazli Dizman, Cristiane D. Bergerot, Alexander Chehrazi-Raffle, Charles B. Nguyen, Abhishek Tripathi, Regina Barragan-Carrillo, Sumanta Kumar Pal

**Affiliations:** 1 Department of Medical Oncology & Therapeutics Research, City of Hope Comprehensive Cancer Center, Duarte, CA 91010, USA; koshah@coh.org (K.U.S.); dacastro@coh.org (D.V.C.); xiaoli@coh.org (X.L.); miguel.zugman@einstein.br (M.Z.); vabreudeges@coh.org (V.A.d.G.); pzang@coh.org (P.D.Z.); skylarreid20@gmail.com (S.R.); teebropaul@gmail.com (T.P.); jaya.goud@winsor.edu (J.G.); samueldickter@gmail.com (S.D.); leadickter@gmail.com (L.D.); lilyml@uci.edu (L.L.); ruchiag@sas.upenn.edu (R.A.); aaronbtlee@yahoo.com (A.L.); chaudharynasr@gmail.com (N.C.); hebrahim@bidmc.harvard.edu (H.E.); bmercier@coh.org (B.M.); ndizman@mdanderson.org (N.D.); achehraziraffle@coh.org (A.C.-R.); charnguyen@coh.org (C.B.N.); atripathi@coh.org (A.T.); rbarraganc@incan.edu.mx (R.B.-C.); 2 The University of Texas MD Anderson Cancer Center, Houston, TX 77030, USA; 3 Oncoclínicas & Co, Medica Scientia Innovation Research (MEDSIR), São Paulo 01309-001, Brazil; cristiane.bergerot@oncoclinicas.com

**Keywords:** bladder cancer, urothelial carcinoma, world bank ranking, clinical trial, high-income countries, non-high-income countries, access, disparities

## Abstract

**Background**: Bladder cancer disproportionately affects non-high-income countries, yet clinical trials underrepresent global diversity. We assessed global availability of bladder cancer trials, their alignment with disease burden, and barriers to equitable care. **Methods**: We queried ClinicalTrials.gov for adult bladder cancer trials from June 2019 to June 2024, excluding observational and non-oncologic trials. Trial characteristics were summarized descriptively, and country data came from the Global Cancer Observatory. Countries were classified per World Bank Ranking (WBR) into high-income (HICs), upper middle-income (UMICs), lower middle-income (LMICs), and low-income countries (LICs). Trials were categorized as HIC-only, non-HIC, or mixed-income trials. Fisher’s exact and Kruskal–Wallis tests compared groups. Multivariable logistic regression assessed associations between trial availability and WBR, national health expenditure, and gross national income (GNI). Univariable linear regression and ANOVA assessed the association between the mortality-to-incident ratio and WBR. **Results**: Of 611 trials, 75.1% were HIC-only, 16.9% non-HIC, and 8.0% mixed-income trials. Non-HIC trials were mainly academic-sponsored (80.6%), while all mixed-income trials had pharmaceutical sponsorship (*p* < 0.001). Non-HIC trials had lower enrollment, less pharmaceutical funding, fewer multinational collaborations, and fewer basket, multi-arm, early-phase designs (all *p* < 0.001). Mixed-income trials were larger, led by HICs, had broader eligibility criteria, more novel therapies, and more frequent use of overall survival endpoints. Trial availability was lower in UMICs (*p* = 0.011), LMICs (*p* = 0.024), and absent in LICs, and positively associated with higher national health expenditure (*p* = 0.007) and GNI (*p* = 0.001). **Conclusions**: Bladder cancer trials remain concentrated in HICs. Mixed-income trials expand access in non-high-income countries, but are exclusively led by HICs and require balanced sponsorship, early-phase research, and lasting local benefits.

## Simple Summary

Bladder cancer affects patients worldwide, but access to clinical trials that test new treatments is not evenly distributed. Many trials are conducted in wealthier countries, even though a large share of the disease burden occurs in lower-resource settings. In this study, we examined where bladder cancer trials are being conducted globally and how this aligns with need. We found that most trials take place in high-income countries, with very limited access in lower-income countries and none in the lowest-income countries. Trials that included multiple countries tended to offer broader access but were often led by wealthier nations. These gaps may limit access to new therapies and reduce the extent to which results apply to diverse populations. Expanding trials to underrepresented regions could improve equity, strengthen global research, and ensure that advances in care benefit patients everywhere.

## 1. Introduction

Bladder cancer is the ninth most diagnosed malignancy worldwide, with 614,000 new cases and 220,000 deaths in 2022. Mortality trends, less affected by diagnostic variability, better reflect disease impact [1]. When viewed by income classification or World Bank Ranking (WBR), mortality burden falls disproportionately on non-high-income countries, while high-income countries (HICs) report higher incidence. Southern European nations such as Spain, Greece, and Italy have the highest incidence of bladder cancer, whereas mortality peaks in northern Africa, namely Egypt, Tunisia, and Libya [2]. Non-high-income countries comprise 56.3% of global bladder cancer mortality in adults aged 20–85 [2], yet have fewer resources to address this growing challenge [3,4]. By the year 2040, chemotherapy and surgery demands for bladder cancer will increase by 66% [5,6,7], while non-high-income countries face deficits in systemic therapy, surgery, and diagnostics [8,9,10,11].

Oncology clinical trials are essential for advancing treatment and reducing mortality, but lack global diversity and remain dominated by HICs [12]. Despite efforts by groups such as the American Society of Clinical Oncology to broaden global participation in cancer trials, data specific to bladder cancer remain scarce [13,14]. One genitourinary-focused study found mortality-to-incidence ratios are highest in less developed countries across testis, prostate, kidney, and bladder cancer, reinforcing the need for clinical trials in these settings [15]. Herein, we evaluate how country income classification correlates with bladder cancer trial availability, trial alignment with global disease burden, and trial characteristics to identify barriers to equitable research.

## 2. Materials and Methods

This retrospective study identified adult bladder cancer trials registered on ClinicalTrials.gov from 1 June 2019 to 1 June 2024 using advanced search filters using the terms “bladder cancer”, “urothelial cancer/carcinoma”, and “upper tract urothelial cancer/carcinoma”. Duplicate records were identified based on unique ClinicalTrials.gov identifiers (NCT numbers) and removed prior to analysis. Trials were included if completed, open to accrual, or ongoing. Exclusions were pediatric, non-interventional, non-oncologic, or trials without patients with bladder cancer. A standardized form was used to capture trial characteristics and design such as diagnosis, sponsorship, phase, purpose, endpoints, sites, etc. Nine authors performed data abstraction under the supervision of DVC and KUS, with all extracted data subsequently reviewed by two investigators for quality control to ensure consistency and accuracy.

Of 194 member states of the United Nations recognized by the World Health Organization (WHO) [16], 178 are listed in the Global Cancer Observatory registry, excluding those lacking WHO data or classified as regions. Author MZ collected each country’s WBR, gross national income (GNI) in USD, national health expenditure, incidence, and mortality. Countries were classified by income per WBR as follows: high-income countries (HICs), upper middle-income countries (UMICs), lower middle-income countries (LMICs), or low-income countries (LICs). Trials were then grouped into three categories by the income classification of participating countries as follows: HIC-only trials (conducted exclusively in HICs), non-HIC trials (conducted exclusively in UMICs and/or LMICs), or mixed-income trials (conducted in both HICs and non-high-income countries). No bladder cancer trials were found in LICs. For multinational trials, the trial was assigned to a single category based on the income classification of all participating countries.

Descriptive statistics summarized trial characteristics. Fisher’s exact test compared categorical variables across HIC-only, non-HIC, and mixed-income trials, while the Kruskal–Wallis test compared continuous variables. Multivariable logistic regression evaluated associations between trial availability and WBR, national health expenditure, gross national income (GNI), and incidence. Specifically, we performed incidence-adjusted normalization (trials per incidence) and a negative binomial regression model with an offset for incidence as a sensitivity analysis to account for disease burden. Univariable linear regression and ANOVA assessed the association between mortality-to-incident ratio and WBR. Significance was defined as a *p*-value < 0.05. Data were collated in Microsoft Excel (version 2210) and analyzed in R (version 4.3.3).

## 3. Results

**Figure 1:**
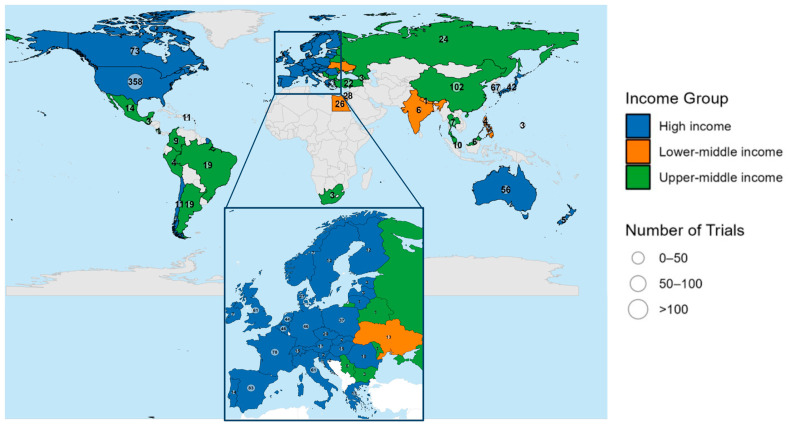
Bladder Cancer Trials by Country and Income Level. Clinical trial availability across countries and income classification per World Bank Ranking. Countries are shaded by income level: dark blue represents high-income countries, green represents upper-middle income countries, and orange represents lower-middle income countries. Circles represent the number of trials per country, with the size of the circle proportional to the number of trials per country according to the legend to the right. A magnified view of Europe highlights smaller details. Countries in white/light grey had no bladder cancer trials.

Of 659 clinical trials identified, 611 trials met inclusion criteria across 62 countries: 75.1% HIC-only trials, 16.9% non-HIC trials, and 8.0% were mixed-income trials. Distribution by country and WBR is seen in Figure 1. All mixed-income trials were led by principal investigators (PIs) based in HICs. Key differences in trial characteristics and design across these three groups are outlined in Table 1 and Table 2.

### 3.1. Sponsorship and Trial Design

Academic institutions were the most common sponsors (54.7%), followed by pharmaceutical companies (pharma) (36.5%), with significant variation by income group (*p* < 0.001). HIC-only trials were primarily academic (54.7%) or pharma-sponsored (34.0%). All mixed-income trials were pharma-sponsored, and non-HIC trials were predominantly academic (80.6%) with minimal pharma sponsorship (17.5%). The presence of pharma funding mirrored these sponsorship patterns (*p* < 0.001).

Trials were predominantly early-phase (I/II, 61.7%) with 13.7% late-phase (III/IV) and 24.6% undefined. Early-phase studies were most common among HIC-only (67.1%) and least among mixed-income trials (42.9%) (*p* < 0.001). Most trials (77.9%) occurred in a single country, with non-HIC trials having the least multinational participation (1 trial). Mixed-income trials had the broadest reach (median of 16 countries per trial, range 2–33; *p* < 0.001) versus HIC-only (1, range 1–15) and non-HIC (1, range 1–2) trials. Enrollment was largest in mixed-income trials (mean of 542 patients, SD 364; *p* < 0.001) versus HIC-only (151, SD 366) and non-HIC (136, SD 248) trials. Multi-arm designs were most common in mixed-income (81.6%) versus HIC-only (55.5%) and non-HIC (51.4%, *p* < 0.001) trials. Basket designs appeared in 24.9% of trials, mainly in HIC-only (29.2%), while non-HIC trials were the most bladder cancer-specific (90.3%, *p* < 0.001). Non-HIC trials enrolled the fewest patients with metastatic disease (16.5%, *p* < 0.001) versus HIC-only (42.9%) and mixed-income (51.0%) trials. Trials including variant histologies were rare (8%). Trials including upper-tract urothelial carcinoma were more frequent in mixed-income (30.6%, *p* < 0.001) than HIC-only (10.9%) or non-HIC (1.0%) trials.

### 3.2. Primary Purpose and Endpoints

The primary purpose of trials varied significantly by income group (Table 2). Most trials evaluated drug therapy (71.0%); all mixed-income trials were drug-focused without any other purpose. Immunotherapy use was highest in mixed-income (73.5%) and lowest in non-HIC trials (36.9%, *p* < 0.001). Surgical trials were most common among non-HIC (32.0%) versus HIC-only (12.0%, *p* < 0.001) trials. Radiation (7.2% vs. 2.9%, *p* = 0.043) and preventative/supportive care trials (14.8% vs. 6.8%, *p* < 0.001) were more frequent among HIC-only than non-HIC trials. Overall survival (OS) as either a primary or secondary endpoints were absent from 61.2% of trials. OS was most often included in mixed-income (83.7% overall, 18.4% primary endpoint) and least in non-HIC (30.1% overall, 2.9% primary, *p* < 0.001) trials. Surrogate endpoints predominated in mixed-income (79.6%) and non-HIC (50.5%) trials, whereas HIC-only trials favored ‘patient-related’ endpoints (45.3%).

### 3.3. World Bank Ranking and Trial Availability

Multivariable logistic regression showed that higher income classification was associated with greater bladder cancer trial availability. Compared to HICs, odds of a trial were lower in UMICs (odds ratio [OR] 0.35, 95% confidence interval [CI] 0.08–0.56, *p* = 0.011) and LMICs (OR 0.16, 95% CI 0.03–0.79, *p* = 0.024); no trials were observed in LICs. Greater GNI (OR 1.00, 95% CI 1.00–1.01, *p* = 0.001) and national health expenditure (OR 1.52, 95% CI 1.12–2.05, *p* = 0.007) as a percentage of gross domestic product (GDP) were also associated with greater trial presence. Each 1% increase in GDP allocated to health spending was associated with a 52% increase in the odds of a trial.

**Figure 2:**
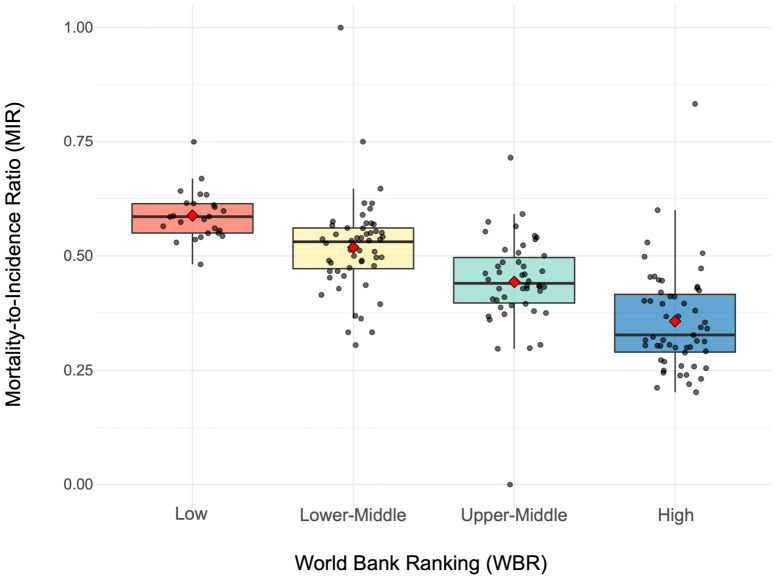
Bladder Cancer Mortality/Incidence Ratio (MIR) across World Bank Ranking. Association between World Bank income classification and bladder cancer mortality-to-incidence ratio (MIR). Higher income groups demonstrated progressively lower MIRs compared with low-income countries (*p* < 0.001). Univariable linear regression showed significant model fit (R^2^ = 0.387, *p* < 0.001). Red diamonds indicate group means; horizontal lines within boxes represent medians.

To assess whether bladder cancer burden differed across income settings, we evaluated the association between World Bank income classification and country-level bladder cancer mortality-to-incidence ratio (MIR) using univariable linear regression with income group modeled as a categorical predictor and high-income countries serving as the reference category. Figure 2 displays the distribution of MIR values across income groups, demonstrating progressively lower MIRs in higher-income countries (*p* < 0.001), consistent with lower mortality relative to incidence.

To further account for differences in underlying disease burden, a negative binomial regression model with an offset for bladder cancer incidence was performed as a sensitivity analysis. After adjustment, World Bank income classification remained significantly associated with trial counts. Compared with HICs, lower-middle-income countries had substantially lower incidence-adjusted trial rates (rate ratio [RR] 0.23, 95% CI 0.11–0.48, *p* = 0.0001), and upper-middle-income countries also had lower rates (RR 0.43, 95% CI 0.23–0.81, *p* = 0.009). These findings were consistent with the primary logistic regression results and support persistent disparities in bladder cancer clinical trial distribution even after accounting for disease burden.

## 4. Discussion

To our knowledge, this is the first global analysis of bladder cancer trial availability and characteristics by income classification. Higher World Bank Ranking, national health expenditure, and GNI were strongly associated with trial availability, reinforcing that HICs dominate the trial landscape as seen across other malignancies [12,17]. Despite accounting for most bladder cancer mortality [2], non-high-income countries remain underrepresented—85.2% of countries without trials were non-high-income. UMICs and LMICs had significantly lower odds of having trials, and LICs had zero. Similar gaps exist in breast, lung, colon, and gastrointestinal cancers [18,19], likely due to limited infrastructure, patient barriers, regulatory complexity, and insufficient funding [14,17,20], factors that may be compounded by economic realities. Although HICs incur higher per-patient cancer costs, non-high-income countries are projected to bear nearly 50.0% of the global economic burden by 2050 [3] despite having fewer resources. While the GNI trial availability OR was close to 1, the narrow CI and *p*-value indicate a meaningful association. Even modest differences in GNI were associated with trial presence, especially when comparing countries with large GNI gaps. These findings suggest an association between economic capacity in healthcare and trial availability, and the need to invest in cancer care, prevention, and research infrastructure in resource-limited settings.

Oncology trials often fail to study cancers with the highest global disease burden [12]. Our findings are consistent with this pattern in bladder cancer, where the trial distribution, inclusion criteria, endpoints, and study purpose appear to be misaligned with populations most affected. Non-high-income countries bear higher bladder cancer mortality-to-incidence ratios [15], likely due to advanced presentation and poorer outcomes [21,22]. However, only 16.9% of trials occurred exclusively in non-high-income countries, enrolling the fewest metastatic and upper-tract urothelial carcinoma cases (<0.001). Variant histologies, more common in LMICs like Egypt and Iran [23,24], were included in only 5.8% of non-HIC trials. OS endpoints, the oncology gold standard [25,26,27], were underutilized across all trials. However, this should be interpreted in the context of trial phase distribution, as the majority of included studies were early-phase and therefore not designed to evaluate survival outcomes. Mixed-income and non-HIC trials more frequently relied on surrogate endpoints, which may expedite completion but limit insight into long-term survival rates [28,29]. The primary purpose of trials may not be fully aligned with the treatment of bladder cancer in non-high-income countries. While surgery remains the most common bladder cancer treatment, 71.0% of trials focused on drug therapy. All mixed-income trials were pharma-funded and drug-focused, wth less emphasis on research in surgery, radiotherapy, palliative care, and prevention [11]. Non-HIC trials had the highest surgery focus (32%, *p* < 0.001), which may reflect surgery’s central role in lower-resource settings, given the cost of oncologic care [30,31]. However, this benefit did not extend to the non-high-income countries in mixed-income trials. Such patterns suggest a potential disconnect between trial design and population need, particularly in non-high-income countries.

Addressing this disconnect requires understanding the characteristics and limitations of non-HIC trials. Unlike prior analyses of other malignancies across income settings, including breast, lung, and gastrointestinal cancers [12], our study found that bladder cancer trials were mainly academia-sponsored. However, non-HIC trials received less pharma and government support than HIC-only trials. Barriers such as limited site capacity, weak regulatory oversight, poor patient protections, lack of insurance, and socio-cultural factors influencing healthcare access and trial participation likely deter pharma engagement [10,14,32]. Expanding trials in non-high-income countries requires greater public, philanthropic, and pharma investment aligned with local needs. With drug access limitations and fewer than half of new cancer drugs delivering meaningful benefit [33,34], research should prioritize population-specific needs over market agendas. Non-HIC trials showed smaller enrollment, fewer multinational collaborations, and less use of basket, multi-arm, or early-phase designs—patterns seen across global oncology research [12,18]. These infrastructure constraints stem from reduced funding, patient access, and provider research training in resource-limited settings [35]. These factors may limit participation in multinational trials, where requirements for imaging, regulatory oversight, and research infrastructure can pose additional barriers in lower-resource settings. While basket and multi-arm trials can streamline drug development, their utility in non-high-income countries remains limited without improved drug access [36]. Non-HIC trials used the fewest immunotherapy agents and were least likely to be early-phase, restricting safety and efficacy testing in diverse populations. As immunotherapy becomes central to bladder cancer treatment, its underrepresentation in non-HIC trials reinforces the need for targeted investment and global collaborations to achieve equitable research.

Mixed-income trials, pharma-funded, and led by HIC-based PIs, had the highest enrollment, use of OS endpoints, and use of immunotherapy with the broadest representation of upper-tract, metastatic, and variant histology disease. However, they were least likely to be early-phase and focused solely on drug therapy. While mixed-income trials may expand research in non-high-income countries by leveraging pharma and HIC support, ethical concerns remain regarding “research parachutism” [37,38] and limited downstream benefit of therapies [20,35], along with other incentives tied to HIC-led trials [32,39]. Their large enrollments, driven by lower costs and faster accrual in non-high-income countries [20,40,41], lead mixed-income trials to be later-phase [17], restricting early-phase innovation to HICs. Exclusive pharma funding and heavy immunotherapy emphasis raise concerns about downstream access for non-high-income countries involved. Policies should ensure mixed-income trials align with local needs through regulatory safeguards, public health prioritization, and generic cancer medicine support [36]. While mixed-income trials appear better matched to the global population in need, we need to determine whether they deliver real-world benefits to patients in non-HICs.

### Limitations

This study has several limitations. First, our analysis relied on a single registry (ClinicalTrials.gov), which may introduce registry selection bias. Trials conducted in non-HICs may be registered in alternative platforms such as the WHO International Clinical Trials Registry Platform or regional/national registries, potentially leading to underestimation of trial availability outside HICs. Additionally, limitations in data capture or reporting may have contributed to the absence of trials in LICs, rather than reflecting a true lack of research activity. As such, our findings should be interpreted as reflective of patterns within this registry rather than the complete global trial landscape. Second, trial availability, defined as the presence of trials active per country, is an imperfect proxy for access and may not accurately capture true patient participation. The absence of patient-level data further limits our ability to assess disparities in enrollment, treatment delivery, and clinical outcomes. Third, registry-based data may be incomplete or inconsistently reported. Manual data extraction may also introduce misclassification, especially when disease stage or endpoints are not clearly specified. Fourth, the exclusion of observational studies may underestimate research activity in lower-resource settings.

Other limitations include the relatively short study period (2019–2024), which may not capture long-term trends. Country classification using WBR income groups may oversimplify heterogeneity within a country. Furthermore, grouping UMIC and LMICs into a single non–high-income category may obscure important distinctions between these settings, although this approach was necessary to preserve statistical power given small subgroup sample sizes. WBR data were based on 2022 Global Cancer Observatory estimates and may not reflect recent economic changes. Finally, incomplete data on trial completion, outcomes, and publications limit real-world impact assessment. Observed associations between trial availability and country-level economic indicators (e.g., GNI and health expenditure) may be influenced by unmeasured confounding factors, including research infrastructure, regulatory environments, and established clinical research networks. This study does not directly evaluate structural and contextual barriers to trial access, including regulatory, logistical, and socio-cultural factors, as these variables are not captured within registry-based datasets such as ClinicalTrials.gov.

## 5. Conclusions

Higher World Bank Ranking, national health expenditure, and gross national income were associated with greater bladder cancer trial availability, concentrating research in HICs despite higher mortality-to-incidence ratios in non-high-income countries. Trial design is often misaligned with populations most affected; non-HIC trials lacked early-phase designs, robust sponsorship, and reflected constrained research infrastructure. Mixed-income trials, leveraging pharma and HIC-based support, were larger with strong endpoints and novel therapies. However, they require balanced sponsorship, early-phase design, and intent to sustainably benefit local health systems. Future work should assess whether mixed-income trials improve outcomes, drug access, and scientific contributions in non-high-income countries.

## Figures and Tables

**Table 1:** Bladder Cancer Clinical Trial Characteristics.

	Overall (N = 611)	HIC-Only Trials (N = 459)	Mixed-Income Trials (N = 49)	Non-HIC Trials (N = 103)	*p*-Value
Sponsor					<0.001
Academia ^1^	334 (54.7%)	251 (54.7%)	0 (0%)	83 (80.6%)	
Combination	20 (3.3%)	19 (4.1%)	0 (0%)	1 (1.0%)	
Government ^2^	34 (5.6%)	33 (7.2%)	0 (0%)	1 (1.0%)	
Pharmaceutical	223 (36.5%)	156 (34.0%)	49 (100%)	18 (17.5%)	
Pharmaceutical Funding					<0.001
No	368 (60.2%)	284 (61.9%)	0 (0%)	84 (81.6%)	
Yes	243 (39.8%)	175 (38.1%)	49 (100%)	19 (18.4%)	
Trial Phase					<0.001
Early (I/II)	377 (61.7%)	308 (67.1%)	21 (42.9%)	48 (46.6%)	
Late (III/IV)	84 (13.7%)	44 (9.6%)	28 (57.1%)	12 (11.7%)	
Not Defined	150 (24.6%)	107 (23.3%)	0 (0%)	43 (41.7%)	
Metastatic Disease					<0.001
No	372 (60.9%)	262 (57.1%)	24 (49.0%)	86 (83.5%)	
Yes	239 (39.1%)	197 (42.9%)	25 (51.0%)	17 (16.5%)	
Multinational					<0.001
No	476 (77.9%)	374 (81.5%)	0 (0%)	102 (99.0%)	
Yes	135 (22.1%)	85 (18.5%)	49 (100%)	1 (1.0%)	
Number of countries/Trial					<0.001
Mean (SD)	2.64 (4.96)	1.54 (1.65)	16.3 (8.86)	1.01 (0.0985)	
Median [Min, Max]	1.00 [1.00, 33.0]	1.00 [1.00, 15.0]	16.0 [2.00, 33.0]	1.00 [1.00, 2.00]	
Planned Enrollment (patients)					<0.001
Mean (SD)	180 (364)	151 (366)	542 (364)	136 (248)	
Median [Min, Max]	74.0 [2.00, 6450]	63.0 [2.00, 6450]	542 [18.0, 1410]	70.0 [6.00, 1600]	
Missing	2 (0.3%)	2 (0.4%)	0 (0%)	0 (0%)	
Variant Histology ^4^					0.361
No	562 (92.0%)	422 (91.9%)	43 (87.8%)	97 (94.2%)	
Yes	49 (8.0%)	37 (8.1%)	6 (12.2%)	6 (5.8%)	
Upper-tract Inclusive					<0.001
No	545 (89.2%)	409 (89.1%)	34 (69.4%)	102 (99.0%)	
Yes ^3^	66 (10.8%)	50 (10.9%)	15 (30.6%)	1 (1.0%)	
Involving Immunotherapy					<0.001
No	332 (54.3%)	254 (55.3%)	13 (26.5%)	65 (63.1%)	
Yes	279 (45.7%)	205 (44.7%)	36 (73.5%)	38 (36.9%)	
Basket Trial Design					<0.001
No	459 (75.1%)	325 (70.8%)	41 (83.7%)	93 (90.3%)	
Yes	152 (24.9%)	134 (29.2%)	8 (16.3%)	10 (9.7%)	
Number of Arms/Trial					<0.001
1	263 (43.0%)	204 (44.4%)	9 (18.4%)	50 (48.5%)	
2	232 (38.0%)	165 (35.9%)	20 (40.8%)	47 (45.6%)	
3+	116 (19.0%)	90 (19.6%)	20 (40.8%)	6 (5.8%)	

^1^ By grant, university, institution; ^2^ By NIH, NCI, Department of Justice; ^3^ Includes cancer of ureter, urethra, renal pelvis in addition to bladder; ^4^ Small cell, squamous cell, adenocarcinoma, mixed variants, or other variant bladder cancer histology.

**Table 2:** Bladder Cancer Clinical Trial Endpoints and Primary Purpose.

	Overall (N = 611)	HIC-Only Trials (N = 459)	Mixed-Income Trials (N = 49)	Non-HIC Trials (N = 103)	*p*-Value
Overall Survival Primary or Secondary Endpoint					<0.001
No	374 (61.2%)	294 (64.1%)	8 (16.3%)	72 (69.9%)	
Yes	237 (38.8%)	165 (35.9%)	41 (83.7%)	31 (30.1%)	
Overall Survival Primary Endpoint					<0.001
No	584 (95.6%)	444 (96.7%)	40 (81.6%)	100 (97.1%)	
Yes	27 (4.4%)	15 (3.3%)	9 (18.4%)	3 (2.9%)	
‘Surrogate’ Primary Endpoint ^1^					<0.001
No	339 (55.5%)	278 (60.6%)	10 (20.4%)	51 (49.5%)	
Yes	272 (44.5%)	181 (39.4%)	39 (79.6%)	52 (50.5%)	
‘Patient-Related’ Primary Endpoint ^2^					<0.001
No	365 (59.7%)	251 (54.7%)	39 (79.6%)	75 (72.8%)	
Yes	246 (40.3%)	208 (45.3%)	10 (20.4%)	28 (27.2%)	
‘Other’ Primary Endpoint ^3^					<0.001
No	449 (73.5%)	332 (72.3%)	48 (98.0%)	69 (67.0%)	
Yes	162 (26.5%)	127 (27.7%)	1 (2.0%)	34 (33.0%)	
Preventative or Supportive Care ^4^					<0.001
No	536 (87.7%)	391 (85.2%)	49 (100%)	96 (93.2%)	
Yes	75 (12.3%)	68 (14.8%)	0 (0%)	7 (6.8%)	
Surgical					<0.001
No	523 (85.6%)	404 (88.0%)	49 (100%)	70 (68.0%)	
Yes	88 (14.4%)	55 (12.0%)	0 (0%)	33 (32.0%)	
Drug Therapy					<0.001
No	177 (29.0%)	136 (29.6%)	0 (0%)	41 (39.8%)	
Yes	434 (71.0%)	323 (70.4%)	49 (100%)	62 (60.2%)	
Radiation					0.043
No	575 (94.1%)	426 (92.8%)	49 (100%)	100 (97.1%)	
Yes	36 (5.9%)	33 (7.2%)	0 (0%)	3 (2.9%)	
Basic Science					0.072
No	603 (98.7%)	453 (98.7%)	49 (100%)	101 (98.1%)	
Yes	8 (1.3%)	6 (1.3%)	0 (0%)	2 (1.9%)	
Radiology					0.487
No	589 (96.4%)	441 (96.1%)	49 (100%)	99 (96.1%)	
Yes	22 (3.6%)	18 (3.9%)	0 (0%)	4 (3.9%)	

^1^ Surrogate endpoints include progression-free survival, disease-free survival, metastasis-free survival, recurrence-free survival, event-free survival, or overall response rate. ^2^ Patient-related endpoints include endpoints such as safety or health-related quality of life. ^3^ Other endpoints include surgical, anesthesia-related, radiologic, biochemical, implementation, or diagnostic. ^4^ Includes screening, supportive care, and surveillance.

## Data Availability

All data supporting the findings of this study are available within the paper. Initial data were collected from ClinicalTrials.gov and the Global Cancer Observatory; the latter can be accessed through https://gco.iarc.who.int/today/ (accessed on 10 February 2025).

## Abbreviations

The following abbreviations are used in this manuscript:

WBR	World Bank Ranking
HICs	High-income countries
UMICs	Upper middle-income countries
LMICs	Lower middle-income countries
LICs	Low-income countries
GNI	Gross national income
WHO	World Health Organization
PIs	Principle investigators
OS	Overall survival

## References

[1] Bray F., Laversanne M., Sung H., Ferlay J., Siegel R.L., Soerjomataram I., Jemal A. (2024). Global cancer statistics 2022: GLOBOCAN estimates of incidence and mortality worldwide for 36 cancers in 185 countries. CA Cancer J. Clin..

[2] Cancer Today. https://gco.iarc.who.int/today/.

[3] Chen S., Cao Z., Prettner K., Kuhn M., Yang J., Jiao L., Wang Z., Li W., Geldsetzer P., Bärnighausen T. (2023). Estimates and Projections of the Global Economic Cost of 29 Cancers in 204 Countries and Territories from 2020 to 2050. JAMA Oncol..

[4] World Health Organization (2018). Pricing of Cancer Medicines and Its Impacts.

[5] Wilson B.E., Jacob S., Yap M.L., Ferlay J., Bray F., Barton M.B. (2019). Estimates of global chemotherapy demands and corresponding physician workforce requirements for 2018 and 2040: A population-based study. Lancet Oncol..

[6] Perera S.K., Jacob S., Wilson B.E., Ferlay J., Bray F., Sullivan R., Barton M. (2021). Global demand for cancer surgery and an estimate of the optimal surgical and anaesthesia workforce between 2018 and 2040: A population-based modelling study. Lancet Oncol..

[7] Scilipoti P., Moschini M., Li R., Lerner S.P., Black P.C., Necchi A., Rouprêt M., Shariat S.F., Gupta S., Morgans A.K. (2024). The Financial Burden of Localized and Metastatic Bladder Cancer. Eur. Urol..

[8] Metzler I., Bayne D., Chang H., Jalloh M., Sharlip I. (2020). Challenges facing the urologist in low- and middle-income countries. World J. Urol..

[9] Raykar N.P., Yorlets R.R., Liu C., Greenberg S.L.M., Kotagal M., Goldman R., Roy N., Meara J.G., Gillies R.D. (2015). A qualitative study exploring contextual challenges to surgical care provision in 21 LMICs. Lancet.

[10] Alemayehu C., Mitchell G., Nikles J. (2018). Barriers for conducting clinical trials in developing countries- a systematic review. Int. J. Equity Health.

[11] Yadav H., Shah D., Sayed S., Horton S., Schroeder L.F. (2021). Availability of essential diagnostics in ten low-income and middle-income countries: Results from national health facility surveys. Lancet Glob. Health.

[12] Wells J.C., Sharma S., Del Paggio J.C., Hopman W.M., Gyawali B., Mukherji D., Hammad N., Pramesh C.S., Aggarwal A., Sullivan R. (2021). An Analysis of Contemporary Oncology Randomized Clinical Trials From Low/Middle-Income vs High-Income Countries. JAMA Oncol..

[13] Wilson B.E., Sullivan R., Peto R., Abubakar B., Booth C., Werutsky G., Adams C., Saint-Raymond A., Fleming T.R., Lyerly K. (2023). Global Cancer Drug Development—A Report From the 2022 Accelerating Anticancer Agent Development and Validation Meeting. JCO Glob. Oncol..

[14] Al Sukhun S.A., Vanderpuye V., Taylor C., Ibraheem A.F., Wiernik Rodriguez A., Asirwa F.C., Francisco M., Moushey A. (2024). Global Equity in Clinical Trials: An ASCO Policy Statement. JCO Glob. Oncol..

[15] Greiman A.K., Rosoff J.S., Prasad S.M. (2017). Association of Human Development Index with global bladder, kidney, prostate and testis cancer incidence and mortality. BJU Int..

[16] Countries Overview | World Health Organization. https://www.who.int/countries.

[17] Jenei K., Moraes F.Y., Gyawali B. (2023). Globalisation of clinical trials in oncology: A double-edged sword?. BMJ Oncol..

[18] Payedimarri A.B., Mouhssine S., Aljadeeah S., Gaidano G., Ravinetto R. (2023). Globalisation of industry-sponsored clinical trials for breast, lung and colon cancer research: Trends, threats and opportunities. BMJ Oncol..

[19] Wells J.C., Fundytus A., Sharma S., Hopman W.M., Del Paggio J.C., Gyawali B., Mukherji D., Hammad N., Pramesh C.S., Aggarwal A. (2022). Randomized Controlled Trials in Lung, Gastrointestinal, and Breast Cancers: An Overview of Global Research Activity. Curr. Oncol..

[20] Rubagumya F., Hopman W.M., Gyawali B., Mukherji D., Hammad N., Pramesh C.S., Zubaryev M., Eniu A., Tsunoda A.T., Kutluk T. (2022). Participation of Lower and Upper Middle–Income Countries in Oncology Clinical Trials Led by High-Income Countries. JAMA Netw. Open.

[21] Nazemi A., Ghodoussipour S., Pearce S., Bhanvadia S., Daneshmand S. (2019). Socioeconomic and insurance status are independent prognostic indicators of higher disease stage and worse prognosis in bladder cancer. Urol. Oncol..

[22] Singla N., Fang D., Su X., Bao Z., Cao Z., Jafri S.M., Xiong G., Zhang L., Hutchinson R., Sagalowsky A. (2017). A Multi-Institutional Comparison of Clinicopathological Characteristics and Oncologic Outcomes of Upper Tract Urothelial Carcinoma in China and the United States. J. Urol..

[23] Naserghandi A., Azizmohammad Looha M., Jameie M., Moradian Haft Cheshmeh Z., Namakin K., Golmakani N., Feyzi A., Shabanipour H., Tofighi Zavareh M.A., Allameh F. (2024). Incidence trends, histological subtypes, and topographical distribution of bladder cancer in Iran: A study based on the Iranian National Cancer Registry during 2006–2015. Front. Oncol..

[24] Martin J.W., Vernez S.L., Lotan Y., Abdelhalim A., Dutta R., Shokeir A., Abol-Enein H., Mosbah A., Ghoneim M., Youssef R.F. (2018). Pathological characteristics and prognostic indicators of different histopathological types of urinary bladder cancer following radical cystectomy in a large single-center Egyptian cohort. World J. Urol..

[25] Driscoll J.J., Rixe O. (2009). Overall survival: Still the gold standard: Why overall survival remains the definitive end point in cancer clinical trials. Cancer J..

[26] Haslam A., Hey S.P., Gill J., Prasad V. (2019). A systematic review of trial-level meta-analyses measuring the strength of association between surrogate end-points and overall survival in oncology. Eur. J. Cancer.

[27] Prasad V., Kim C., Burotto M., Vandross A. (2015). The Strength of Association Between Surrogate End Points and Survival in Oncology: A Systematic Review of Trial-Level Meta-analyses. JAMA Intern. Med..

[28] Chen E.Y., Joshi S.K., Tran A., Prasad V. (2019). Estimation of Study Time Reduction Using Surrogate End Points Rather Than Overall Survival in Oncology Clinical Trials. JAMA Intern. Med..

[29] Booth C.M., Sengar M., Goodman A., Wilson B., Aggarwal A., Berry S., Collingridge D., Denburg A., Eisenhauer E.A., Ginsburg O. (2023). Common Sense Oncology: Outcomes that matter. Lancet Oncol..

[30] Kingham T.P., Alatise O.I., Vanderpuye V., Casper C., Abantanga F.A., Kamara T.B., Olopade O.I., Habeebu M., Abdulkareem F.B., Denny L. (2013). Treatment of cancer in sub-Saharan Africa. Lancet Oncol..

[31] Krishnan M., Agarwal P., Pinninti R., Rajappa S. (2023). Global inequalities in availability of systemic therapies for cancer care and strategies to address them. J. Surg. Oncol..

[32] Glickman S.W., McHutchison J.G., Peterson E.D., Cairns C.B., Harrington R.A., Califf R.M., Schulman K.A. (2009). Ethical and scientific implications of the globalization of clinical research. N. Engl. J. Med..

[33] Tibau A., Molto C., Ocana A., Templeton A.J., Del Carpio L.P., Del Paggio J.C., Barnadas A., Booth C.M., Amir E. (2018). Magnitude of Clinical Benefit of Cancer Drugs Approved by the US Food and Drug Administration. J. Natl. Cancer Inst..

[34] Del Paggio J.C., Sullivan R., Schrag D., Hopman W.M., Azariah B., Pramesh C.S., Tannock I.F., Booth C.M. (2017). Delivery of meaningful cancer care: A retrospective cohort study assessing cost and benefit with the ASCO and ESMO frameworks. Lancet Oncol..

[35] Gyawali B. (2016). Me, Too. J. Glob. Oncol..

[36] Gyawali B., Sullivan R. (2017). Economics of Cancer Medicines: For Whose Benefit?. New Bioeth..

[37] Gyawali B., Carson L.M., Berry S., Moraes F.Y. (2022). Challenges of globalization of cancer drug trials- recruitment in LMICs, approval in HICs. Lancet Reg. Health Am..

[38] Smith J. (2018). Parasitic and parachute research in global health. Lancet Glob. Health.

[39] Ravinetto R., Tinto H., Diro E., Okebe J., Mahendradhata Y., Rijal S., Gotuzzo E., Lutumba P., Nahum A., Nys K.D. (2016). It is time to revise the international Good Clinical Practices guidelines: Recommendations from non-commercial North–South collaborative trials. BMJ Glob. Health.

[40] Qiao Y., Alexander G.C., Moore T.J. (2019). Globalization of clinical trials: Variation in estimated regional costs of pivotal trials, 2015–2016. Clin. Trials.

[41] Perkovic V., Patil V., Wei L., Lv J., Petersen M., Patel A. (2012). Global Randomized Trials: The Promise of India and China. J. Bone Jt. Surg. Am..

